# Maintenance of physicochemical, optical, and biological properties of conventional glass ionomer cement enriched with an anacardic acid-derivative compound

**DOI:** 10.1007/s00784-026-06762-6

**Published:** 2026-03-06

**Authors:** Bruna Genari, Bruna Leis Endres, Erick Rabelo, Luiz Antonio Soares Romeiro, Andressa Souza de Oliveira, Thuy Do, Reem El-Gendy, Vitória Beatriz Souza da Silva, Naile Dame-Teixeira, Fernanda Cristina Pimentel Garcia

**Affiliations:** 1 Restorative Dentistry Department, School of Dentistry, Federal University of Minas Gerais, Belo Horizonte, Brazil; 2 Department of Dentistry, School of Health Sciences, University of Brasilia, Darcy Ribeiro University, Campus Asa Norte, Brasília, Brazil; 3 Laboratory for the Development of Therapeutic Innovations (LDT), Tropical Medicine Center, Faculty of Medicine, University of Brasilia, Brasilia, Brazil; 4 Department of Pharmacy, School of Health Sciences, University of Brasilia, Brasilia, Brazil; 5 Division of Oral Biology, School of Dentistry, University of Leeds, Leeds, UK

**Keywords:** Biomaterials, Anacardic acid, Mechanical properties, Biofilms, Dental caries, Glass ionomer cements

## Abstract

**Objectives:**

To evaluate the physicochemical, optical, and antimicrobial properties of a conventional glass ionomer cement (GIC) modified with anacardic acid (LDT11).

**Materials and methods:**

LDT11, extracted from cashew nutshells, was incorporated into GIC (FX ULTRA, Shofu, USA) at 0.5%, 1%, and 2% (w/w), with 0% as control. Disc-shaped specimens (6–15 mm diameter × 1 mm thickness) were prepared for all evaluations*.* Setting time (ISO 9917; n = 3), acid-base reaction efficiency (FTIR, COO^−^/COOH ratio; n = 3), water sorption and solubility (ISO 4049; n = 5), diffusion coefficient (n = 5), surface roughness (Ra, Rz, Rv; n = 5), and color parameters (CIELab, CIEDE2000; n = 5) were measured. Antimicrobial characterizations were carried out, with discs inoculated with *Streptococcus mutans* UA159 and incubated anaerobically for 7 days (early biofilms) and 14 days (mature biofilms). Biofilms were dyed with the Live/Dead biofilm viability kit and then imaged using confocal laser scanning microscopy (n = 6). Data were analyzed using one-way ANOVA with Tukey post hoc or Kruskal-Wallis with Dwass-Steel-Critchlow-Fligner tests (p < 0.05).

**Results:**

LDT11 incorporation did not significantly affect setting time, acid-base reaction efficiency, solubility, diffusion coefficient, and surface roughness. FTIR spectra revealed no alterations in setting-related functional groups, while LDT11 was identified in the 1000–1100 cm⁻¹ range. At 2%, LDT11 significantly increased water sorption and caused visible color changes (*p* < 0.05). The 0.5% and 1% groups significantly reduced *S. mutans* viability compared to control, with the 1% group exhibiting the most pronounced and sustained effect (*p* < 0.0001).

**Conclusions:**

Incorporation of the anacardic acid derivative up to 1% maintained physicochemical properties of GIC while providing antimicrobial activity.

## Introduction

While many general health conditions, including those impacting oral health, have shown downward trends [1], oral diseases remain highly prevalent, with untreated carious lesions in permanent teeth ranking among the most common global health issues [2]. This shift is accompanied by more teeth being retained in the mouth but affected by conditions such as periodontal disease, increasing exposed root surfaces susceptible to root caries lesions (RCLs) [3, 4].

Glass ionomer cements (GICs) are recognized as materials of choice for restoring teeth in patients with multiple carious lesions, particularly when the oral environment requires adaptation. Their fluoride release, biocompatibility, chemical bonding and mechanical similarity to dentin contributes to the prevention of caries adjacent to fillings and enhances longevity [5]. These features make GIC suitable for subgingival RCLs, shallow wide cavities, and situations with poor moisture control, where the composites perform inadequately [2]. GICs are also less technique-sensitive, facilitating use in older or dependent patients, who are often affected by these lesions [3]. Their simplified clinical application reduces the need for rotary instruments and can also lower treatment costs, thereby improving accessibility [4]. However, restoration failure is frequently due to recurrent caries [6], and RCLs often extend beneath the gingival margin, in contact with inflamed periodontal tissues [7]. In this context, incorporating bioactive compounds with anti-inflammatory and antimicrobial properties into GICs has been proposed as a promising approach to enhance their preventive potential and clinical performance in managing root caries.

Cashew Nut Shell Liquid (CNSL), mainly composed of anacardic acid (AA), shows antioxidant, anti-inflammatory, antimicrobial, and matrix metalloproteinases (MMPs)-inhibitory properties [8, 9], including effects against oral biofilms [10, 11]. A saturated AA derivative, LDT11, has demonstrated antimicrobial potential, low cytotoxicity, and stimulation of pulp stem cells, indicating favorable biocompatibility [12]. LDT11 exerts its antibacterial activity primarily as a surface-active agent, disrupting the native membrane-associated function of integral proteins through physical membrane disruption*.* [11]. This compound also exhibited potent anti-collagenolytic activity, inhibition of *Porphyromonas gingivalis* collagenase reaching up to 96.8% [12, 13]. Given Brazil’s extensive cashew production, CNSL represents a sustainable source for biomedical applications [14]. LDT11 represents a promising additive for restorative materials, potentially reducing gingival inflammation in RCLs. Although its bioactivity has been demonstrated in solution [15], including the ability to alter gene expression within complex oral biofilms, further studies are required to determine whether these properties are preserved when integrated into restorative formulations [15]. 

Although anacardic acid derivatives have been successfully incorporated into other dental materials, such as adhesives [16], their integration into glass ionomer cements represents a novel strategy to enhance bioactivity for root caries management. Anacardic acid exhibited no antibacterial activity against S. mutans at concentrations up to 800 µg/ml [10]. This study evaluated the physicochemical, optical, and antimicrobial properties of a GIC modified with LDT11, testing the null hypothesis that LDT11 incorporation would not alter these properties compared to unmodified GIC*.*

## Materials and methods

### Experimental design

This in vitro study employed a parallel-group design with LDT11 concentration as the primary factor (0%, 0.5%, 1%, 2% w/w). Response variables included setting time, acid-base reaction efficiency, water sorption/solubility, surface roughness, color parameters, and antimicrobial activity. Sample testing was performed by a single trained operator in a blinded manner (group codes masked until analysis). Sample sizes were based according to prior literature [16, 17] to achieve 80% power at α = 0.05*.*

### Anacardic acid

LDT11 chemical design, isolation, and purification were previously described [18]. Briefly, 150 g of cashew nutshell and absolute ethanol (400 mL) were added to a Soxhlet extraction system, under heating for 4 h. The solvent was evaporated, yielding 60 mL of CNSL. In a 50 mL flask, 30 g of CNSL and 15 g of Ca(OH)₂ were added to a mixture of methanol (180 mL) and water (30 mL), under stirring and reflux for 3 h. After cooling to room temperature, the mixture was filtered, and the resulting solid was washed with ethyl acetate, followed by hydrogenation, filtration, solvent remotion and recrystallized steps, yielding 3.5 g of 2-hydroxy-6-pentadecylbenzoic acid (LDT11).

### Incorporation of LDT11 into GIC

LDT11 was incorporated into the conventional GIC powder (Glass Ionomer FX ULTRA, Shofu, USA) at 0.5% (GIC0.5), 1% (GIC1), and 2% (GIC2), in weight using an analytical balance (Bel Engineering, Italy) and mixed for 30 s in a vortex mixer (KASVI Basic, KASVI, Brazil). A control group without LDT11 (GIC0) was maintained.

### Setting time

The initial setting time test was conducted following an adaptation of ISO 9917-1:2007 (n = 3)*.* The powder-to-liquid 1:1 ratio and handling of the material followed the manufacturer’s instructions. Discs (6.0 ± 0.1 mm x 1.0 ± 0.1 mm) were prepared and indented with a modified Gillmore needle (100 ± 0.5 g, 2.0 ± 0.1 mm tip) [19] every 15 s until no visible mark was detected under observation in Zeiss Stemi DR 1040 stereo microscope (Carl Zeiss, Oberkochen, Germany) with 20x magnitude.

### Fourier-transform infrared (FTIR) spectroscopy

Samples were prepared as described in section 2.3 and analyzed immediately after mixing and 6 min after mixing. FTIR spectroscopy was performed using a Perkin-Elmer BX instrument (Perkin-Elmer, USA) coupled with a diamond crystal of an attenuated total reflection (ATR) device. The scans were recorded between 2000 –1000 cm-^1^ (16 scans), as previously [20].

The efficiency of the acid-base reaction was assessed using the intensity (peak height) of COO- (1600 cm^− 1^) and COOH ($\:\sim$1700 cm^−1^) and 1600 cm^− 1^/1700 cm^− 1^ ratio as described by TALAL et al. (2009) [21]. Spectra from GIC0 and GIC2 at both initial and final time points were compared and subtracted to identify LDT11.

### Water sorption and solubility

Tests followed ISO 4049:2000 (*n* = 5). Discs (15 ± 0.1 mm x 1 ± 0.1 mm) were produced. The protective paste from material kit was applied until the initial setting time was completed. The samples were stored in a desiccator at 37 °C and weighed repeatedly using an analytical balance until a constant mass (m_1_) was obtained – defined as no variation greater than *± *0.0001 g over 24 h. Diameter and thickness of each specimen were measured with a digital caliper to calculate the volume (V) of each disk. The specimens were individually immersed in sealed glasses vials containing 10 mL of distilled water at 37 °C for seven days. After, disks were gently wiped with an absorbent paper, weighed one minute later to determine m_2_, and returned to desiccator. To obtain a constant mass (m_3_), the specimens were weighed consecutively, following the same protocol used to m_1_. Water sorption (WS) and solubility (WL) were calculated using the following formulae: *WS = (m*_*2*_ *- m*_*3*_*)/V (1)*; and *WL = (m*_*1*_ *- m*_*3*_*)/V (2)*. The diffusion coefficient (%) was obtained by summing the sorption and solubility values.

### Surface roughness

Surface roughness (Ra, Rz, Rv) was measured using a roughness tester (SJ-201, Mitutoyo, Japan) on surface of GIC discs (6.0 ± 0.1 mm x 1.0 ± 0.1, *n* = 5) after 24 h at 37 ± 1 °C. Discs were polished using a standardized protocol: sequential wet grinding with 600-, 800-, and 1200-grit silicon carbide papers, followed by ultrafine diamond paste (1 μm) on a felt wheel at 300 rpm for 30 s per step*.* Three equidistant measurements taken along 2.5 mm at a speed of 0.25 mm/s were averaged.

### Color parameters

Color was measured with a spectrophotometer (VITA Easyshade V, VITA Zahnfabrik, Germany), using CIElab and CIEDE2000 systems. Color differences were calculated by comparing groups without LDT11 to those with each concentration of the compound (*n* = 5). The CIELab color difference was determined using the equation:

$$\triangle E_{ab}=\left[\left(\triangle L\right)^2+\left(\triangle a\right)^2+\left(\triangle b\right)^2\right]^\frac12.$$

Similarly, the CIEDE2000 color difference was calculated using the following formula:

$$\begin{array}{c}\triangle E_{00}=\left\{\left[\frac{\triangle L'}{\left(k_LS_L\right)}\right]^2+\left[\frac{\triangle C'}{\left(k_CS_C\right)}\right]^2+\left[\frac{\triangle H'}{\left(k_HS_H\right)}\right]^2\right.\\+\left.R_T\left[\frac{\triangle C'}{\left(k_CS_C\right)}\right]X\left[\frac{\triangle H'}{k_H}S_H\right]\right\}^\frac12,\end{array}$$

where $\:\varDelta\:$L’, $\:\varDelta\:$C’, and $\:\varDelta\:$H’ represent the differences in lightness, chroma, and hue. S_L_, S_C_, and S_H_ are the weighing functions for lightness, chroma, and hue components, respectively, while K_L_, K_C_, and K_H_ are parametric factors that adjust for different viewing conditions, which were set to 1.

### Antimicrobial assay

Sterilized GIC discs with or without LDT11 were inoculated with *Streptococcus mutans* UA159 and incubated anaerobically for 7 days (early biofilms) and 14 days (mature biofilms). The groups comprised triplicates with concentrations of control 0%, 0.5%, and 1% of LDT11. After incubation period, biofilms were dyed with Filmtracer Live/Dead Biofilm Viability Kit (Molecular Probes, Inc.) for 30 min at room temperature, as previously described [12] and then imaged using Confocal Laser Scanning Microscopy (Leica Microsystems), using a dry lens of 20 × and 1.5 zoom. 3D biofilm images were generated using Leica Application Suit X software, in duplicates in each sample, resulting in *n* = 6 per group, showing both 3D and the maximum projection option 2D images. Cell viability was calculated as a percentage of live/dead using the Biofilm Viability Checker tool in Leica Application Suite X software, with parameters including automatic thresholding for green (live, SYTO 9) and red (dead, propidium iodide) fluorescence channels, minimum cell area of 1 μm², and calculation of live/dead ratio as the proportion of green pixel area to total fluorescent area across 2D maximum projections.

### Statistical analysis

Normality was verified using Shapiro-Wilk. One-way ANOVA and Tukey post hoc was applied to reaction efficiency, surface roughness, solubility, color parameters, and antimicrobial assay. Kruskal-Wallis and Dwass-Steel-Critchlow-Fligner was used for setting time and sorption. A significance level of 5% was adopted for all tests.

## Results

**Fig. 1:**
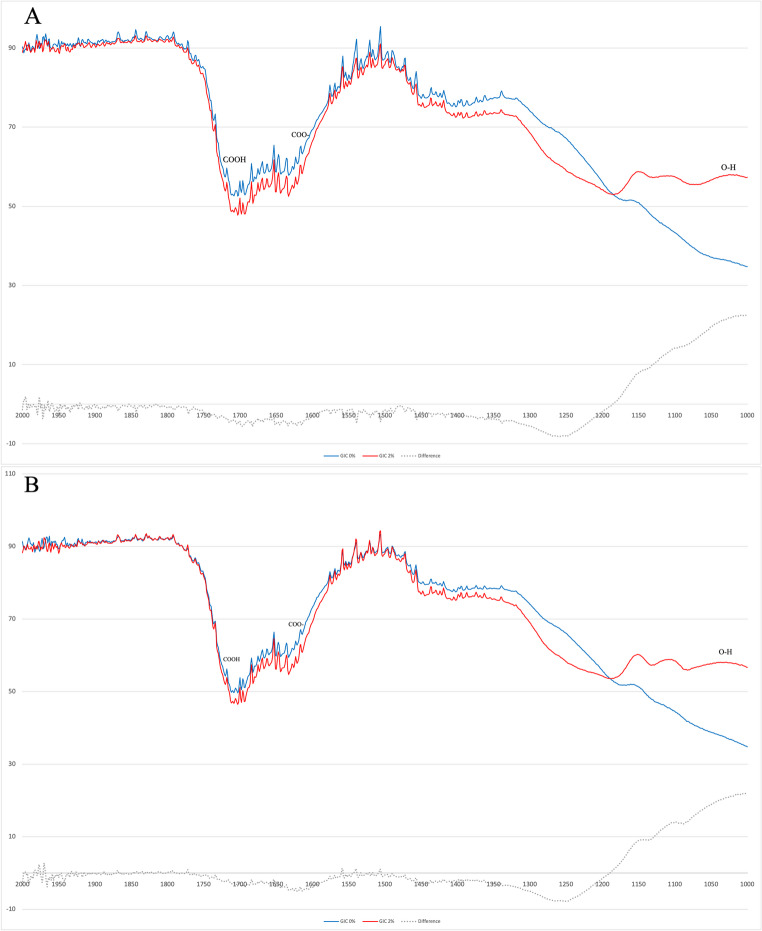
FTIR spectra of LDT11 into GIC. (**A**) Initial FTIR spectra of GIC without LDT11 (GIC 0%, blue line), and enriched with LDT11 (GIC 2%, red line), measured immediately after mixing. The figure also presents the spectral differences between the samples with and without LDT11 (dotted gray), highlighting the peak in the 1000–1100 cm^− 1^ range. (**B**) Final FTIR spectra of GIC 0% (blue line), and GIC 2% (red line), measured after 6 min, along with the corresponding spectral differences (dotted gray), emphasizing the peak in the 1000–1100 cm− 1 region

The setting time ranged from 4.84 $\:\pm\:\:$0.58 to 5.92 $\:\pm\:\:$0.29 min. LDT11 did not significantly affect setting time (*p* = 0.05), and acid-base reaction efficiency of GIC (Table 1). The presence of LDT11 was detected in the 1000–1100 cm^−1^ range (Fig. 1). The COO- at 1600 cm⁻¹ and COOH around 1700 cm⁻¹ functional groups involved in the setting reaction were not influenced by LDT11, as indicated by the absence of changes in their respective peaks before and after setting (Fig. 1A and B, respectively). The addition of 2% LDT11 significantly increased sorption compared to the 0%, 0.5%, and 1% concentrations (Table 1). However, the solubility and diffusion coefficient of the groups containing LDT11 did not significantly differ from the control group.

**Table 1:** Setting time (minutes, min), acid-base reaction efficiency, sorption (µg/mm^3^), solubility (µg/mm^3^), and diffusion coefficient (%)

Groups	Setting time (min)	Acid-base reaction efficiency	Sorption (µg/mm^3^)	Solubility (µg/mm^3^)	Diffusion coefficient (%)
GIC 0%	4.84$\:\pm\:$0.58^A^	0.81$\:\pm\:$0.02^A^	16.60$\:\pm\:$26.80^A^	-1.65$\:\pm\:$39.00^A^	8.62$\:\pm\:$28.00^A^
GIC 0.5%	5.08$\:\pm\:$0.14^A^	0.95$\:\pm\:$0.07^A^	28.60$\:\pm\:$35.50^A^	-25.90$\:\pm\:$17.30^A^	16.60$\:\pm\:$15.90^A^
GIC 1%	5.33$\:\pm\:$0.14^A^	1.13$\:\pm\:$0.10^A^	27.90$\:\pm\:$48.20^A^	-20.50$\:\pm\:$27.70^A^	24.70$\:\pm\:$25.00^A^
GIC 2%	5.92$\:\pm\:$0.29^A^	1.11$\:\pm\:$0.08^A^	82.80$\:\pm\:$42.70^B^	-25.10$\:\pm\:$44.80^A^	57.70$\:\pm\:$68.10^A^

Mean ± standard deviation. Different uppercase letters in the same column indicate statistically significant differences (*p* < 0.05)

Surface roughness results are presented in Table 2. The incorporation of LDT11 at different concentrations did not significantly affect surface roughness for any parameter (Ra, Rz, Rv) (*p* = 0.74). As LDT11 concentration increased into GIC, a greater color difference was observed (Table 2). Specifically, the addition of 2% LDT11 resulted in a significantly higher color difference compared to the group with 0.5% LDT-11, for $\:\varDelta\:$L, $\:\varDelta\:$E_ab_ and $\:\varDelta\:$E_00_.

**Table 2:** Surface roughness (Ra, Rz, Rv) and color parameters (∆L, ∆a, ∆b, ∆Eab, ∆E00)

Groups	Surface roughness (µm)			∆L	∆a	∆b	∆Eab	∆E00
	Ra	Rz	Rv					
GIC 0%	0.45$\:\pm\:$0.09^A^	3.65$\:\pm\:$0.74^A^	2.62$\:\pm\:$0.63^A^	–	–	–	–	–
GIC 0.5%	0.35$\:\pm\:$0.07^A^	3.73$\:\pm\:$0.99^A^	2.96$\:\pm\:$0.90^A^	–6.60 ± 1.20^A^	1.84 ± 1.62^A^	1.14 ± 1.60^A^	7.21 ± 1.41^A^	4.90 ± 0.93^A^
GIC 1%	0.39$\:\pm\:$0.13^A^	3.81$\:\pm\:$0.75^A^	2.99$\:\pm\:$0.53^A^	–8.52 ± 2.66^AB^	2.48 ± 2.66^A^	0.08 ± 2.42^A^	9.38 ± 2.91^AB^	6.50 ± 2.00^AB^
GIC 2%	0.39$\:\pm\:$0.07^A^	3.45$\:\pm\:$0.06^A^	2.59$\:\pm\:$0.40^A^	–13.46 ± 4.79^B^	0.92 ± 0.46^A^	–1.02 ± 1.56^A^	13.59 ± 4.85^B^	9.98 ± 3.84^B^

Mean ± standard deviation. Different uppercase letters in the same column indicate statistically significant differences (*p* < 0.05)

**Fig. 2:**
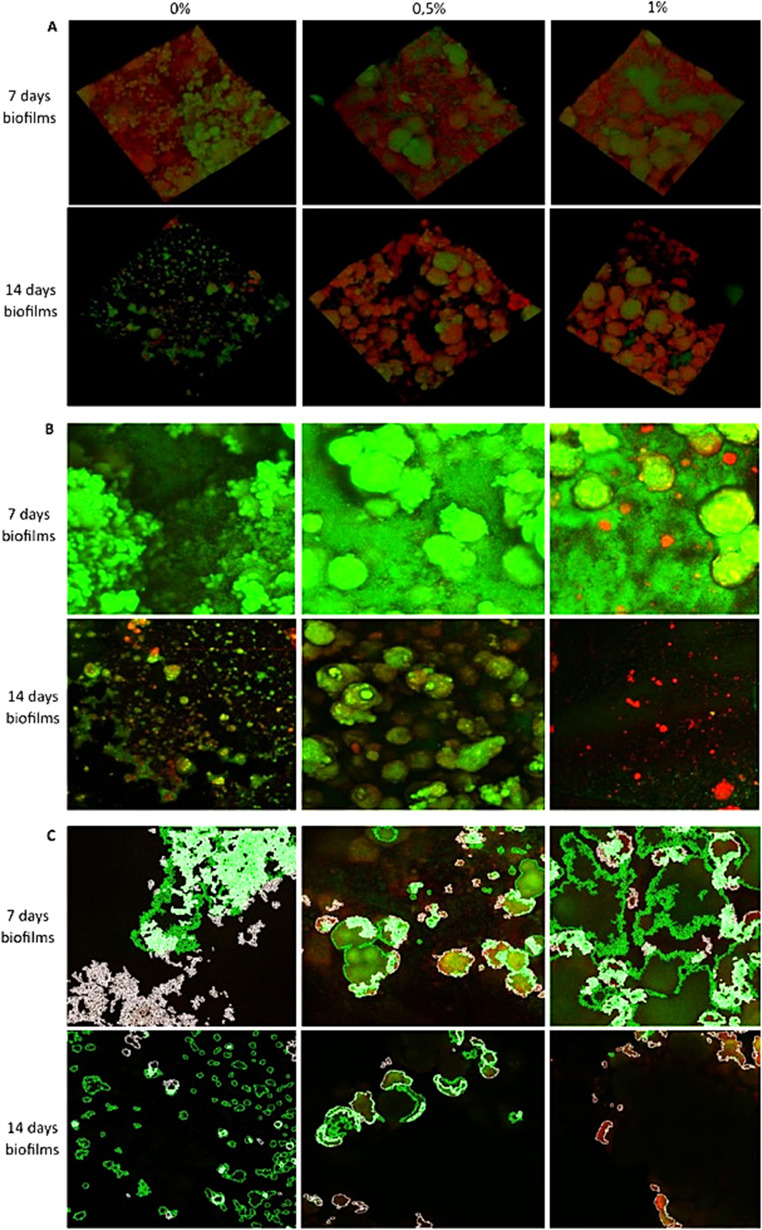
Confocal microscopy images of 7- and 14-day biofilms. The groups comprised triplicates with concentrations of control 0%, 0.5%, and 1% of LDT11. (**A**) 3D Confocal images showing the surface of the biofilms. (**B**) Maximum projection images showing all biofilm layers combined and condensed in 2D. (**C**) Representative images of cell identification in the biofilm viability checker

**Fig. 3:**
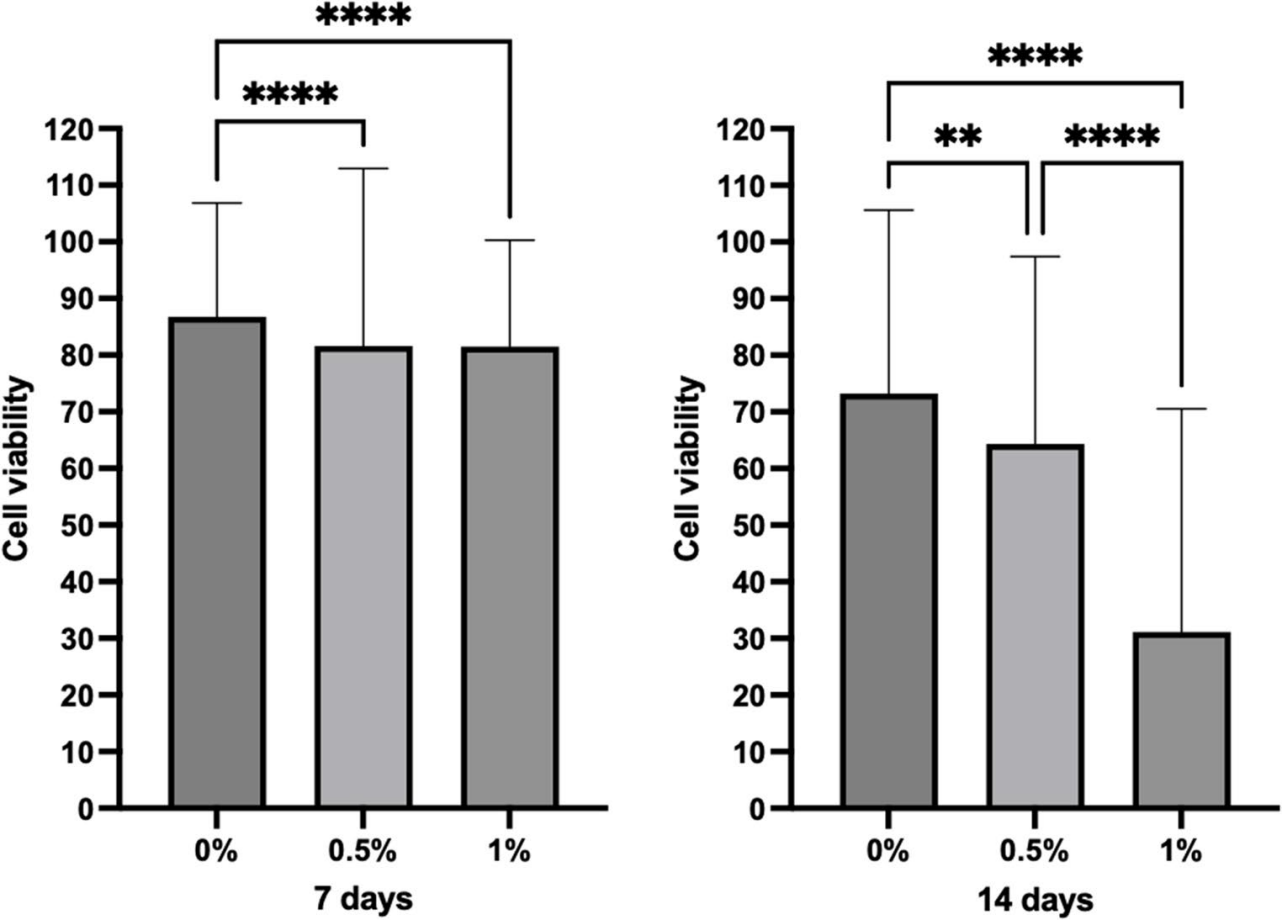
Cell viability *Streptococcus mutans* biofilms at 7 and 14 days of incubation over GIC enriched or not with LDT11 using confocal laser scanning microscopy. Significant differences between groups are indicated: **p < 0.01, ***p < 0.001, ****p < 0.0001

The antimicrobial activity assay showed that LDT11 exhibited a more pronounced effect on the biofilm at 14 days than at 7 days in the 3D images (Fig. 2A). Although no substantial difference was detected at the earlier time point, the effect becomes clearer in the 2D projection images shown in Fig. 2B particularly for the 1%-LDT11 concentration. The 3D images mainly capture the biofilm’s surface layers, which may explain the limited visibility of changes at 7 days, whereas the 2D projections provide a more complete view through the entire biofilm thickness. To quantify these differences, we applied the Biofilm Viability Checker algorithm. As shown in Fig. 2C, the algorithm’s output highlights individual cells with circles, enabling the calculation of the proportion of live and dead cells through color-based differentiation. In the 7-day biofilm, the 0.5% and 1% groups had lower *S. mutans* viability than the 0% control group (*p* < 0.0001). By the 14-day, the viability was lower than in 7 days, confirming sustainability of the antimicrobial effect over time. The difference between the control 0% with 0.5% persisted, and the 1% group exhibited significantly lower cell viability than the other two groups (*p* < 0.0001) (Fig. 3).

## Discussion

This study presents a novel approach to enhance the bioactivity properties of conventional GIC. LDT11, a hemi-synthetic compound with properties such as anti-inflammatory, biocompatibility with dental pulp stem cells, anti-collagenase, antimicrobial against planktonic Gram-positive and Gram-negatives as well as against unispecies and complex biofilms [12, 15]. These characteristics are particularly relevant given the clinical application of GIC in managing RCLs. To the best of our knowledge, this is the first study incorporating anacardic acid into GIC at concentrations 0.5 to 2% (5,000–20,000 $\:{\upmu\:}\mathrm{g}/\mathrm{m}\mathrm{l}$*)* [20]. Here we show that the incorporation of LDT11 into the GIC maintained its antimicrobial activity without adversely affecting its physicochemical properties.

LDT11 incorporation did not compromise the acid-base setting reaction, as evidenced by the setting time, reaction efficiency, and FTIR spectra. The GIC containing LDT11 remained within the setting time limit of 6.0 min, as specified by ISO standards (ISO 9917-1:2007). The higher concentrations of COOH in a salt compound (around 1700 cm^− 1^) and a reduced number of ionized COO- (1500–1600 cm^− 1^ range) groups [21, 22] are found after the setting reaction. Although the additional carboxylic group in LDT11 might theoretically accelerate the acid-base reaction, this was not observed, likely due to its limited solubility and steric hindrance from the hydrophobic 15-carbon alkyl chain, which may restrict ion diffusion and interaction with the glass surface [23, 24]. This supports the observed maintenance of setting time within ISO limits. Similar to observations in anacardic acid derivatives where the hydrophobic side chain modulates activity [11], the alkyl chain in LDT11 likely hinders full participation in the acid-base reaction. The FTIR analysis supported the setting time findings, with no interference of LDT11 in the 1600 cm⁻¹ range, corresponding to the asymmetric stretching of carboxylate groups (COO⁻), a key marker of the acid-base reaction forming metal carboxylate salts (Ca²⁺/Al³⁺) in conventional GICs [21, 22]. A comparison of spectra from GIC2 and GIC0, both before (Fig. 1A) and after setting (Fig. 1B), highlights a peak in the 1000–1100 cm^− 1^ range. This region corresponds to a broad band attributed to out-of-plane O-H bending vibration, while C-O stretching vibration in acids appears near 1260 cm^− 1^ [25]. The characterization of the acid-base reaction with LDT11 in chemically cured GIC thereby paves the way for its potential incorporation into resin-modified GIC in future studies.

The chemical composition of materials influences their physicochemical properties. The presence of hydroxyl and carboxylic groups in the AA molecule increases water affinity, leading to higher water sorption [26, 27]. The 2% LDT11 concentration demonstrated this effect after 7 days of immersion in distilled water, which is a sufficient period to observe such changes in restorative materials [17]. These interactions are particularly relevant given the moisture present in the oral environment. Although the type of storage solution may influence sorption and solubility, no significant differences have been reported between distilled water and artificial saliva [17]. Increased water sorption leads to greater spacing between chains, resulting in volume expansion and potential chain breakage, which can cause maladaptation or impair the mechanical performance of material [28]. This finding is especially important for GIC, a material known for its water absorption capacity, reinforcing the need for surface protection [29]. Despite the increase in sorption, solubility was not affected by the addition of LDT-11. The maintenance of mass per volume, indicating no material degradation, suggests structural stability of GIC even with LDT-11, thereby supporting its potential to maintain the longevity of restorations under water immersion conditions [27, 30]. Although anacardic acid contains chemical groups capable of forming hydrogen bonds with water [31], the low concentrations of LDT11 in GIC was not sufficient to significantly increase water permeation, as confirmed by the diffusion coefficient. Another factor contributing to the unchanged diffusion coefficient between groups is that LDT11 did not interfere with the cross-linking reactions during setting [31]. The surface roughness of restorative materials is a crucial factor for microbial retention [32]. A threshold Ra value of 0.2 $\:{\upmu\:}$m has been established to prevent bacterial accumulation [33]. Clinically, these roughness values (Ra < 0.5 μm) are below thresholds associated with increased plaque retention (0.7–1.4 μm) [34], suggesting that LDT11-enriched GIC would not promote microbial accumulation and could support long-term restoration integrity and color stability [35]. Finishing and polishing procedures can influence surface roughness [36]. Using the conditions of the present study, GIC without LDT11 exhibited a Ra value of 0.45 ± 0.09 $\:{\upmu\:}$m (Table 2). LDT11 incorporation did not increase surface roughness, which may indicate its structural stability among intermolecular chains, preventing particle loss during the finishing and polishing procedures [37], contributing to preservation of surface smoothness. Poor esthetics is a known drawback of GIC restorations. Compared to control group, the addition of 2% LDT11 significantly affected color parameters. A significant reduction in lightness was observed at the 2% LDT11 concentration. Given the observed ∆E values exceeded 3.3, a noticeable visual difference is expected [38]. In the case of ∆E₀₀, the perceptibility and acceptability thresholds are defined as values of 0.81 and 1.77, respectively [39]. Based on these thresholds, LDT11 incorporation resulted visually perceptible differences (Table 2), particularly evident to trained professionals. Therefore, the perceptible color changes at 2% LDT11 (∆E > 3.3, ∆E00 > 1.77) may limit its use in esthetically demanding anterior restorations; however, for root caries in less visible areas, such as subgingival lesions, this may be clinically acceptable, prioritizing bioactivity over esthetics*.* The CIEDE2000 formula provides a better fit than CIELAB when assessing color differences [39, 40], as it incorporates corrections for the non-uniformity of the CIELAB color space. These include weighting function (*S*_*L*_,*S*_*C*_,*S*_*H*_), a rotation term (*R*_*T*_)-which primarily affects low chroma colors-and parametric factors (*k*_*L*_,*k*_*C*_,*k*_*H*_) that adjust for illumination and visual perception effects [40]. These refinements make CIEDE2000 a more reliable indicator of human perceptibility, reinforcing its adoption in dentistry in recent years [41]. Due to significantly higher water sorption and noticeable color changes in the 2% LDT11-containing GIC, this concentration was excluded from the antimicrobial assay. A dose-response effect was observed in the viability of *S. mutans* biofilms when grown on LDT11-enriched GIC. These findings indicate that LDT11 retains its antimicrobial efficacy even when incorporated into a restorative material, suggesting that integration of LDT11 into GIC formulations may represent a sustainable and bioactive alternative for root caries restorative procedures. The local antimicrobial activity provided by the LDT11-enriched GIC over a 15-day period can be particularly beneficial in patients with active lesions extended under gingival margin. In cases of root caries, especially when the lesion is located subgingivally and accompanied by gingival bleeding [13], this initial antimicrobial effect may contribute to a reduction in local inflammation, thereby improving the periodontal environment. This short-term therapeutic window is often sufficient to stabilize the site and allow for subsequent placement of a more definitive restoration. In such clinical scenarios, a rapid initial response during the first two weeks is essential to enhance treatment outcomes and patient recovery, particularly in individuals with high caries risk and compromised oral health. The sustained antimicrobial effect correlates with LDT11’s mechanism of membrane disruption [11], reducing S. mutans viability in mature biofilms. Clinically, this could improve outcomes in high-risk patients with subgingival root caries by minimizing recurrent caries and inflammation. From a cost-benefit perspective, sourcing LDT11 from sustainable cashew nut shell offers an economical additive, potentially reducing treatment failures and associated healthcare costs without requiring new manufacturing processes*.* Further studies are needed to determine whether these effects can persist beyond the two-week period. Given the complex oral environment and microbiota associated with root caries particularly the lesions that extend subgingivally [13], there is a pressing need to enhance the properties of existing restorative materials and to develop innovative technologies for lesions management. In vitro design restricts the direct extrapolation of results to clinical practice. Nevertheless, beyond its potential use in root caries lesions, this GIC may be beneficial in managing avulsion cases resulting from dental trauma, as a strategy to prevent root resorption - an application that warrants further investigation. Given the promising results observed, it is important to assess the material’s properties under conditions that simulate the oral environment. Factors such as pH cycling, thermal fluctuations, and aging can significantly affect surface integrity. Moreover, acid resistance is a key consideration, as GICs can undergo varying levels of degradation in acidic settings*.*

## Conclusion

The incorporation of LDT11 at different concentrations did not significantly alter the setting time, acid-base reaction efficiency, solubility, diffusion coefficient, or surface roughness of conventional GIC. However, the 2% LDT11 concentration significantly increased water sorption. Higher LDT11 concentrations also led to a progressive increase in color difference, with the 2% group exhibiting notable changes. A dose-response effect was observed in the viability of *S. mutans* biofilms, and the incorporation of 1% LDT11 demonstrated antimicrobial activity while maintaining key physicochemical properties of conventional GIC*.*

## Data Availability

No new data were generated or analyzed in support of this research. All findings is included in the manuscript.
